# Comparative genome analysis of plant ascomycete fungal pathogens with different lifestyles reveals distinctive virulence strategies

**DOI:** 10.1186/s12864-021-08165-1

**Published:** 2022-01-07

**Authors:** Yansu Wang, Jie Wu, Jiacheng Yan, Ming Guo, Lei Xu, Liping Hou, Quan Zou

**Affiliations:** 1 School of Electronic and Communication Engineering, Shenzhen Polytechnic, 518000 Shenzhen, P. R. China; 2 Institute of Fundamental and Frontier Sciences, University of Electronic Science and Technology of China, 610054 Chengdu, P. R. China; 3 Key Laboratory of Biotechnology for Medicinal Plants of Jiangsu Province, School of Life Sciences, Jiangsu Normal University, 221116 Xuzhou, P. R. China; 4 Department of Agronomy and Horticulture, University of Nebraska, Lincoln, USA; 5 Beidahuang Industry Group General Hospital, Harbin, China; 6 State Key Laboratory of Tree Genetics and Breeding, Northeast Forestry University, Harbin, China

**Keywords:** Plant fungal pathogens, Carbohydrate-active enzymes, Secondary metabolites, Effector proteins, Gene gain and loss

## Abstract

**Background:**

Pathogens have evolved diverse lifestyles and adopted pivotal new roles in both natural ecosystems and human environments. However, the molecular mechanisms underlying their adaptation to new lifestyles are obscure. Comparative genomics was adopted to determine distinct strategies of plant ascomycete fungal pathogens with different lifestyles and to elucidate their distinctive virulence strategies.

**Results:**

We found that plant ascomycete biotrophs exhibited lower gene gain and loss events and loss of CAZyme-encoding genes involved in plant cell wall degradation and biosynthesis gene clusters for the production of secondary metabolites in the genome. Comparison with the candidate effectome detected distinctive variations between plant biotrophic pathogens and other groups (including human, necrotrophic and hemibiotrophic pathogens). The results revealed the biotroph-specific and lifestyle-conserved candidate effector families. These data have been configured in web-based genome browser applications for public display (http://lab.malab.cn/soft/PFPG). This resource allows researchers to profile the genome, proteome, secretome and effectome of plant fungal pathogens.

**Conclusions:**

Our findings demonstrated different genome evolution strategies of plant fungal pathogens with different lifestyles and explored their lifestyle-conserved and specific candidate effectors. It will provide a new basis for discovering the novel effectors and their pathogenic mechanisms.

**Supplementary Information:**

The online version contains supplementary material available at 10.1186/s12864-021-08165-1.

## Background

Plant pathogens with different lifestyles utilize distinct strategies to interact with their host plants. Necrotrophs such as the white mold fungus *Sclerotinia sclerotiorum* and the gray mold fungus *Botrytis cinerea* often secrete enzymes and toxins to kill their hosts and reproduce in dead tissue [1], while biotrophs such as powdery mildew fungi (e.g., *Blumeria graminis*) establish haustoria and take up nutrients within living host cells [2]. Hemibiotrophs such as *Magnaporthe oryzae* first adopt a biotrophic growth phase and later change into a necrotrophic infection phase [3]. The plastic genome structure of fungal species could help them evolve distinct sets of genes to successfully complete their infection lifestyle [4–6]. In the variable genome compartment, gene gain and loss events reflect high adaptation within pathogenic life histories [7, 8]. Effector genes are often located in genomic compartments with high mutation and/or recombination rates, such as repeat-rich regions, near telomeres, this promotes genes lose, gain, or mutation, and are among the most rapidly evolving genes in pathogen populations [9]. Even the birth-and-death model of an effector gene was proposed by Fouché, et al. [10], whether plant pathogens virulence is associations with their different lifestyles and how does  evolution change the diversity of effector proteins among these species is poorly understood.

Successful plant pathogens have to overcome physical barriers, suppress or manipulate the plant immune system, and retrieve nutrients from host tissues when infecting host plants for pathogenicity [11]. The plant cell wall is an important physical barrier that serves as the first line of plant defense that plant pathogens encounter during infection and is composed of basic carbohydrate ingredients, including cellulose, hemicellulose, pectin and lignin. Plant pathogens produce carbohydrate-active enzymes (CAZymes) that play a pivotal role in breaching the frontline of plant defense [12]. Important members of the CAZyme family are cell wall degrading enzymes (CWDEs), including glycolside hydrolases (GHs), polysaccharide lyases (PLs) and carbohydrate esterases (CEs), that cleave or modify oligo- and polysaccharides as well as other glycoconjugates and are involved in complex carbohydrate metabolism [13]. Filamentous fungi also produce a wide repertoire of bioactive secondary metabolites. Many secondary metabolites act as pathogenicity factors and have detrimental impacts on host health. For example, trichothecenes produced by *Fusarium* spp., T-toxin polyketides produced by *Cochliobolus heterostrophus* and AM-toxin cyclic peptides by *Alternaria alternata* are clearly involved in pathogenicity [14, 15]. Many observations found that fungi have evolved different CAZyme and secondary metabolite production strategies to adapt their own lifestyle [16–18]. Well known functions of melanin are protecting spores or mycelium against external environment, such as desiccation, oxygen and UV. It is also required for appressorium formation of *M. grisea* and allows the fungus invades the plant tissue [19].

Plant pathogens secrete diverse groups of effector proteins to gain virulence advantages. One major role of effectors is to suppress plant immunity and aid infection for pathogenicity and successful establishment of different lifestyles [20–22]. Most effectors have N-terminal signal sequences for secretion, and this feature helps predict the entire inventory of secreted proteins [23–26]. The other trait of effector proteins is that they are rich in cysteine (greater than 2 %), which would help in the formation of stabilizing disulfide bridges [23]. A proportion of effectors perform multifarious functions, such as host cell wall degradation, act as inhibitors of host defensive enzymes (e.g., proteases) or affect defense signaling pathways (e.g., salicylic acid biosynthesis) [27]. They function either in the plant extracellular space (apoplast) or inside plant cells after translocation from the pathogen [28]. Despite their key functions as immunity suppressors, effectors can be recognized by the plant surveillance system, leading to effector-trigger immunity (ETI) in a manner highly specific to resistant (R) proteins [29, 30].

Many known plant pathogens are mostly distributed in four classes in Ascomycota. These phytopathogenic fungi have diverse survival strategies and a high level of ecological diversity [31]. To uncover lifestyle-specific features of effector proteins, we employed a comparative genomic method to analyze 17 selected plant fungal pathogens of Ascomycota, all causing important crop diseases, including 2 biotrophs, 6 necrotrophs, and 9 hemibiotrophs (Tables S1 and S2). Two human pathogens and three biotrophs from Basidiomycota were included for comparison. We focus on the following two aspects: (i) genome structure and evolution characteristics among pathogens with different lifestyles and (ii) whether there are differences in the effector genetic diversity of these plant pathogens. The identification and analyses of the repertoire of effector variants is a prerequisite to understand pathogen-plant interactions and will provide important insights into the different infection strategies of pathogens.

## Results

### Lower gene gain and loss events occurred in ascomycete plant biotrophs

**Fig. 1:**
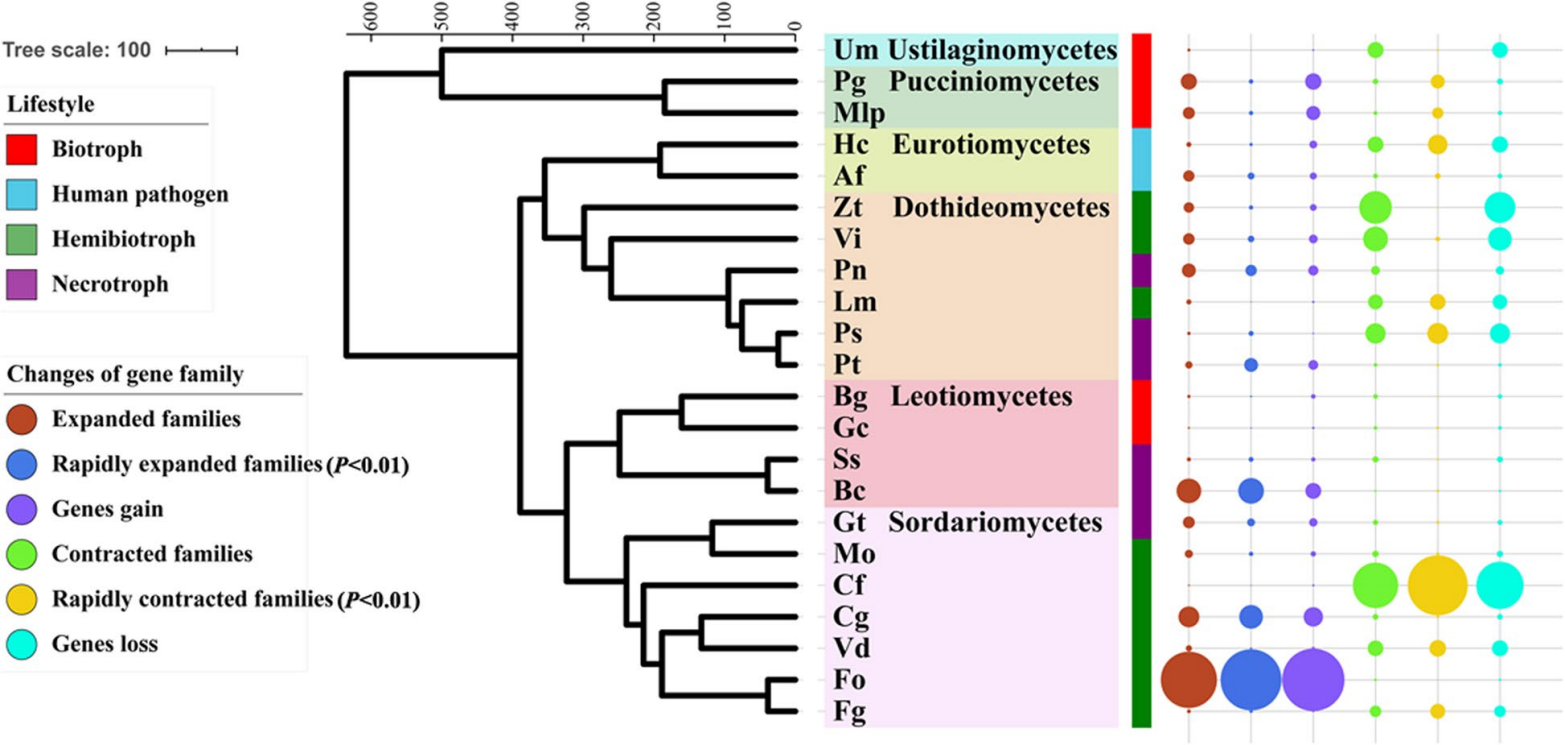
Phylogenetic relationship of pathogens and gene family size changes among these species. The tree was constructed by RAxML based on single-copy gene families present in the fungal pathogens examined. The colored strips represent different lifestyles of fungi. The circles represent the numbers of expanded and contracted families or gain and loss genes of each fungus. The threshold for being defined as rapidly evolving families is set to 0.01

The genome sizes of the 22 genomes ranged between 19.7 and 124.5 Mb (Table S1). The number of protein sequences ranged from 6,532 to 27,347 (Table 1). The ascomycete biotrophs (e.g., *B. graminis* and *Golovinomyces cichoracearum*) have relatively smaller proteomes than those of other species (Table 1). A total of 16,036 protein families were identified in 22 species, among which the number of families expanded and contracted. The two ascomycete biotrophs, *B. graminis* and *G. cichoracearum*, harbor a relatively low number of expanded and contracted families (Fig. 1 and Table S3), implying a slow rate of gene gain and loss in their evolution. Although both species live a hemibiotrophic lifestyle, *Fusarium oxysporum* and *Ceratocystis fimbriata* were in sharp contrast to all species tested. *F. oxysporum* had the most rapidly expanded families (1,222), while *C. fimbriata* contained the largest number of rapidly contracted families (243), indicating that they had undergone significant gene gains or losses in their genomes (Fig. 1 and Table S3). Even though both *F. oxysporum* and *F. graminearum* belong to the genus *Fusarium*, *F. oxysporum* revealed obvious gene family expansion (Fig. 1) because of its broad host adaptation and different reproductive strategies. Ma, et al. [32] discovered that *F. oxysporum* (15 chromosomes) harbors a larger genome assembly than *F. graminearum* (4 chromosomes), and the lineage-specific (LS) genomic regions in *F. oxysporum* account for more than one-quarter of the genome and are rich in transposons and genes with distinct evolutionary profiles but related to pathogenicity, indicative of horizontal acquisition.

**Table 1:** Number of proteins in the proteome, secretome and effectome for each fungus

Phylum	Fungus	Proteome	Secretome	S/P (%)	Effectome	E/P (%)
Ascomycota	*Aspergillus fumigatus*	9630	798	8.29	74	0.77
	*Botrytis cinerea*	13,703	1226	8.95	94	0.69
	*Blumeria graminis*	6835	665	9.73	229	3.35
	*Ceratocystis fimbriata*	7266	566	7.79	60	0.83
	*Colletotrichum gloeosporioides*	15,071	2015	13.37	353	2.34
	*Fusarium graminearum*	13,313	1263	9.49	223	1.68
	*Fusarium oxysporum*	27,347	1800	6.58	337	1.23
	*Golovinomyces cichoracearum*	6532	373	5.71	54	0.83
	*Gaeumannomyces tritici*	14,650	1465	10.00	262	1.79
	*Histoplasma capsulatum*	9313	344	3.69	48	0.52
	*Leptosphaeria maculans*	12,469	1033	8.28	176	1.41
	*Magnaporthe oryzae*	12,989	1727	13.30	422	3.25
	*Parastagonospora nodorum*	15,994	1348	8.43	249	1.56
	*Pyrenophora seminiperda*	8698	620	7.13	86	0.99
	*Pyrenophora teres*	13,126	1102	8.40	181	1.38
	*Sclerotinia sclerotiorum*	14,490	850	5.87	79	0.55
	*Verticillium dahlia*	10,535	1086	10.31	129	1.22
	*Venturia inaequalis*	13,741	1754	12.76	486	3.54
	*Zymoseptoria tritici*	10,963	912	8.32	189	1.72
Basidiomycota	*Puccinia graminis*	15,979	1892	11.84	618	3.87
	*Melampsora larici-populina*	16,372	1781	10.88	562	3.43
	*Ustilago maydis*	6782	600	8.85	88	1.30

S: Secretome; P: Proteome; E: Effectome

### Lower number of CWDE gene homologs in plant biotrophs

**Fig. 2:**
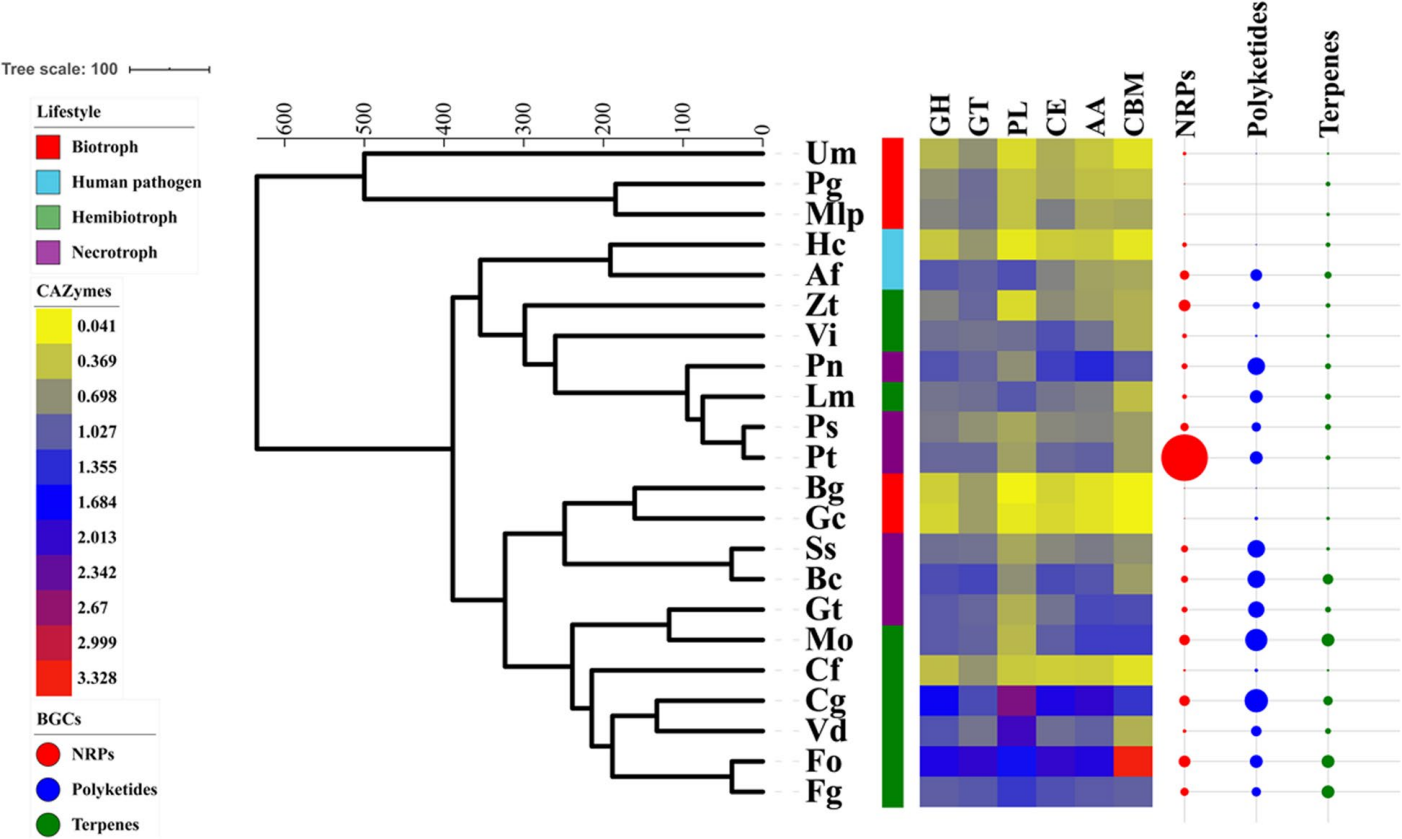
Numbers of CAZyme-encoding genes and secondary metabolite biosynthetic gene clusters in each fungus. The colored strips represent different lifestyles of fungi. The heatmap indicates the number of genes encoding CAZymes involved in GH, GT, PL, AA and CBM activity. The numbers were normalized with the “scale” function in the R “base” package. The circles denote the number of each biosynthetic gene cluster (BGC) type, including nonribosomal peptides (NRPs), polyketides, and terpenes

CAZymes play critical roles in the degradation of the plant cell wall. The CE, GH, and PL superfamilies are also known as cell wall degrading enzymes (CWDEs) due to their role in the disintegration of the plant cell wall by bacterial and fungal pathogens [13]. To better understand the frequency and distribution of CAZyme-coding genes in the genome of each lifestyle, we detected the presence of CAZyme-coding genes using a homology-based approach. We identified lower numbers of three types of CWDEs (GHs, PLs and CEs) in plant biotrophs than in necrotrophs and hemibiotrophs (Fig. 2 and Table S4), consistent with previous studies showing that a lower proportion of CWDEs allows biotrophic organisms to better adapt live plant tissue and avoid plant cell death [16, 21, 33]. A highly complex set of CWDE homologs in the genomes of plant hemibiotrophic and necrotrophic pathogens indicates their significant roles in pathogenicity by being involved in the breakdown of the plant cell wall. However, the human biotrophic pathogen *A. fumigatus* displayed a distinct pattern with plant biotrophs that had higher CWDE gene numbers (Fig. 2 and Table S4). The enzymes for auxiliary activities (AAs) and carbohydrate-binding modules (CBMs) were also reduced in plant biotrophs. These noncatalytic proteins could increase the affinity of enzymes to substrates. Glycosyltransferases (GTs) involved in the biosynthesis of oligosaccharides, polysaccharides, and glycoconjugates are more similar to hemibiotrophic and necrotrophic pathogens.

### Loss of secondary metabolite biosynthesis genes in plant biotrophs

Secondary metabolites (SMs) are low-molecular-weight compounds with a significant ecological, symbiotic or pathogenic role. SM biosynthetic pathways that produce polyketides, nonribosomal peptides (NRPs), ribosomal peptides, terpenes and hybrid metabolites are the most prevalent. Biosynthetic gene clusters (BGCs) are responsible for the production of these small molecules. To investigate the presence and diversity of BGCs involved in specialized metabolism pathways, the fungal genomes were processed by performing antiSMASH analyses. Plant biotrophs possess a contracted array of nonribosomal peptide, polyketide, and terpene biosynthetic gene clusters, implying that they have a weaker capacity to produce these secondary metabolites than necrotrophs and hemibiotrophs (Fig. 2), consistent with biotrophs being able to keep their host alive and take nutrients from it, while necrotrophs need to produce more diverse secondary metabolites to maintain generally broader host ranges than biotrophs [34, 35]. Human pathogens, *H. capsulatum* and ascomycete plant biotrophs have lower numbers of NRPs (4 gene clusters), polyketides (1 gene cluster), and terpenes (4 gene clusters), while *A. fumigatus* has more than several times (8 NRPs, 10 polyketides, 6 terpenes) that of *H. capsulatum* (Table S5), which is inconsistent with plant ascomycete fungal pathogens. Although there was a discrepancy in NRP numbers with Syme, et al. [36], both our results and theirs reflected that NRPs were overrepresented in *P. teres* compared to other ascomycete fungal genomes. The antiSMASH analysis also revealed that the ascomycete genome formed a larger complement of secondary metabolite gene clusters than the basidiomycetes examined in this study even the strains from basidiomycetes harbor larger genome (Fig. 2), the results are similar to a previous report [17].

### Plant biotrophs contain distinctive secretomes and effectomes

**Fig. 3:**
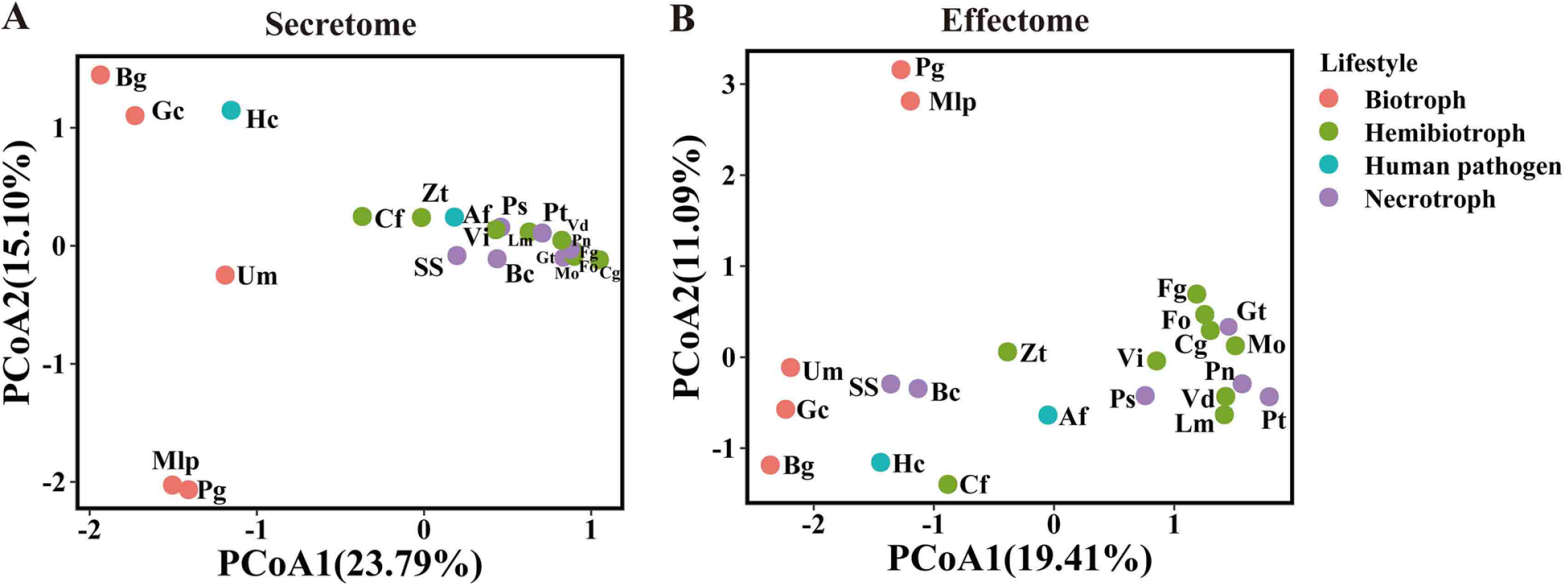
PCoA plots of the secretome (**A**) and effectome (**B**) based on the weighted UniFrac distance metric. Af, *Aspergillus fumigatus*; Bc, *Botrytis cinerea*; Bg, *Blumeria graminis*; Cf, *Ceratocystis fimbriata*; Cg, *Colletotrichum gloeosporioides*; Fg, *Fusarium graminearum*; Fo, *Fusarium oxysporum*; Gc, *Golovinomyces cichoracearum*; Gt, *Gaeumannomyces tritici*; Hc, *Histoplasma capsulatum*; Lm, *Leptosphaeria maculans*; Mo, *Magnaporthe oryzae*; Pn, *Parastagonospora nodorum*; Ps, *Pyrenophora seminiperda*; Pt, *Pyrenophora teres*; Ss, *Sclerotinia sclerotiorum*; Vd, *Verticillium dahlia*; Vi, *Venturia inaequalis*; Zt, *Zymoseptoria tritici*; Pg, *Puccinia graminis*; Mlp, *Melampsora larici-populina* ; Um, *Ustilago maydis*

The predicted secretome and effectome varied in terms of the number of proteins among 22 genomes. The number of predicted secreted proteins ranged from 344 to 2015, or 3.69–13.37 % of their respective proteomes, while the number of predicted effectors ranged from 48 to 618, accounting for 0.52–3.87 % of the proteome, respectively (Table 1). We clustered 1902 orthologous families for secreted proteins and 388 orthologous families for effectors across all species using OrthoFinder. Biotrophs were distinctively separated from the other groups in the secretome and effector with principal coordinate analysis (PCoA) (Fig. 3; ANOSIM: *P*= 0.001 and 0.01, respectively), while hemibiotrophic and necrotrophic pathogens apparently showed an aggregated distribution pattern (Fig. 3; ANOSIM: *P* > 0.1), which indicated the genetic diversity variations of effector and secreted proteins in different lifestyles.

**Fig. 4:**
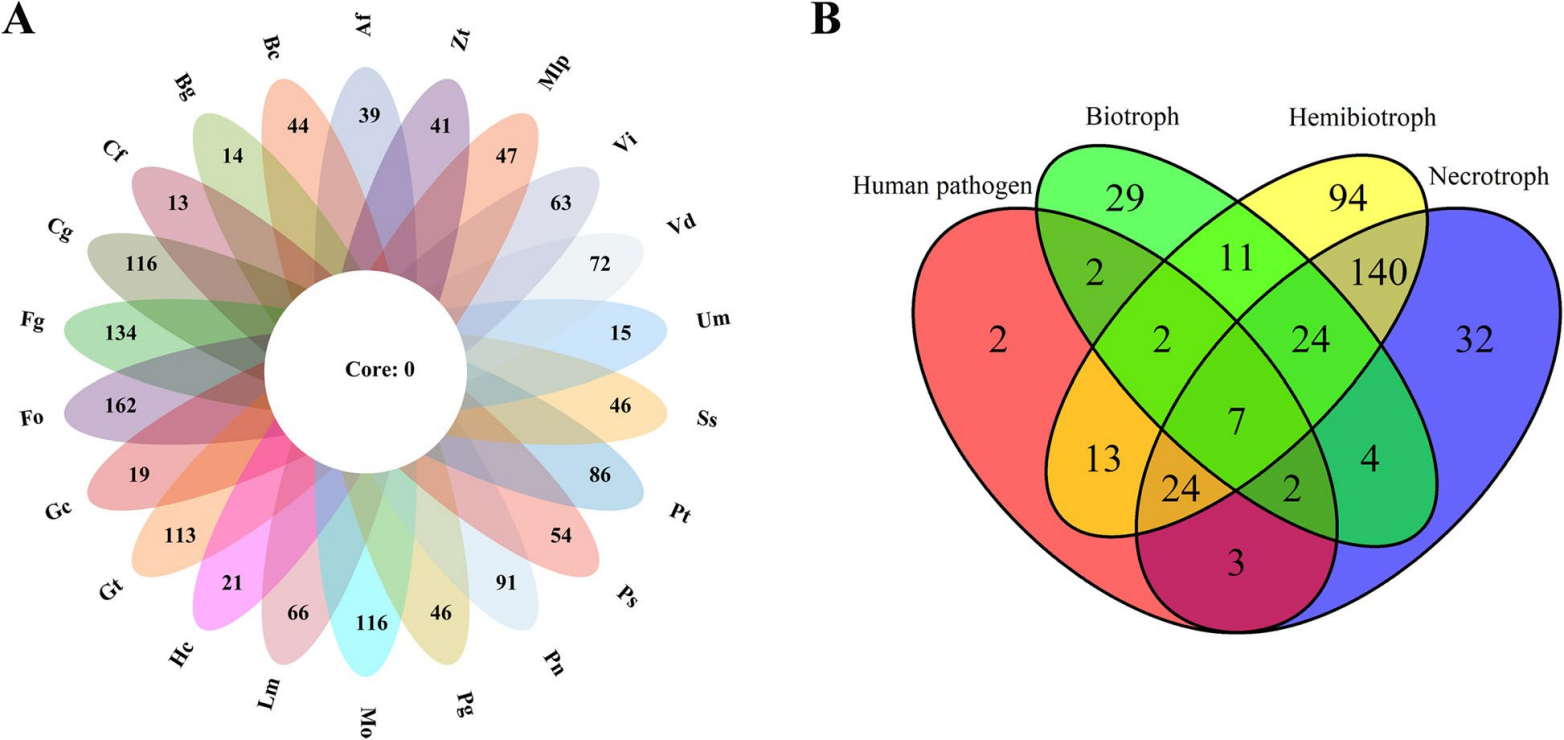
Flower plot (**A**) and Venn diagram (**B**) illustrating core and specific gene families of the effectome among each species or lifestyle. Flower plots showing no overlapping effectors were detected between species. The number in the flower plot represents the total effector gene family of each species. The number in the Venn diagram indicates the core or specific gene family of each lifestyle

To further probe the possible mechanism that causes the distribution pattern of effectors between biotrophs and other organisms, we determined the biotroph-specific and core candidate effector families. Some biotroph-specific candidate effectors were found to be species-specific and often lacked homologs (Table S6). They were annotated to exhibit diverse functions (Table S7). Although no common effectors were detected among species, as illustrated by the flower plot (Fig. 4 A), we defined 7 core effector families from the overlapping sets among different lifestyles (Fig. 4B). Of these, five effector families were annotated as cutinase (OG0000001), endo-1,4-beta-xylanase (OG0000005), FK506-binding protein (OG0000020), cysteine-rich secretory protein (OG0000037) and endosomal P24B protein (OG0000039) (Fig. S1 and Table S8). The domains they contained included cutinase (PF01083.22), Glyco_hydro_11 (PF00457.17), FKBP_C (PF00254.28), CAP (PF00188.26) and EMP24_GP25L (PF01105.24) (Table 2 and Fig. S2). One of the remaining two core effector families contains proteins with unknown function (DUF3455; OG0000043), while the other (OG0000019) is excluded from our analysis due to the lack of observed protein domains.

**Table 2:** Pfam domain annotations of core effector families

Target name	Sequence number	Domain accession	Descriptions
Cutinase	43	PF01083.22	Cutinase (OG0000001)
Glyco_hydro_11	22	PF00457.17	Glycosyl hydrolases family 11 (OG0000005)
FKBP_C	14	PF00254.28	FKBP-type peptidyl-prolyl cis-trans isomerase (OG0000020)
CAP	10	PF00188.26	Cysteine-rich secretory protein family (OG0000037)
EMP24_GP25L	10	PF01105.24	emp24/gp25L/p24 family/GOLD (OG0000039)
DUF3455	9	PF11937.8	Protein of unknown function (DUF3455) (OG0000043)
Homeobox_KN	2	PF05920.11	Homeobox KN domain
DUF2990	1	PF11693.8	Protein of unknown function (DUF2990)
Others	13		No annotation information

### Establishment of the genome database of plant fungal pathogens

To make our data easily available to the research community, we developed a genome database for plant fungal pathogens and encourage researchers to use these resources (http://lab.malab.cn/soft/PFPG). Currently, online resources provide genomic and protein sequences for our data only, but they could be integrated in the future with additional genomic or transcriptomic data from other plant fungal pathogens. The genome hub we built offers a suite of bioinformatics tools to explore, analyze, and interpret these data. For example, we set up sequence search web services that allow users to search their sequence of interest, perform BLAST similarity searches to compare sequences against the genomic and protein sequence database and utilize the genome browsing tool for visualization of the genomic data. Compared to other plant pathogenic fungi genome database, such as FungiDB (https://fungidb.org), our database has accumulated and stored sequences of the secretome and effectome of plant fungal pathogens. The goal of the website is to establish a user-friendly platform for phytopathologists to extract and summarize plant fungal pathogen information intuitively from multiple data sources and to explore the novel virulence factors.

## Discussion

The plasticity of the genetic profile allows biotrophic, necrotrophic and hemibiotrophic lifestyles to form evolutionarily stable life history traits within fungi. Expansion and contraction of gene families is important to pathogen and host coevolution [37, 38]. Our results suggested that all selected pathogens have experienced gene family expansion and contraction, even though two ascomycete biotrophs showed slow gain and loss rates. Compared with biotrophs, necrotrophs and hemibiotrophs experienced relatively obvious turnover of genes as well as intensive dependence on the changes of host-life-state. Host-adapted lifestyles range from mutualistic symbiosis to pathogenic states, the millions of years of co-evolution has resulted a complex molecular dialogue between organisms [39]. Gain and loss of genomic islands can lead to rapid lifestyle transitions in plant-associated *Pseudomonas* [40]. *Cuscuta australis* also experienced remarkably high levels of gene contraction to adapt their parasitic lifestyle [38]. Hence, understanding of the co-evolutionary process in the arms race between plant pattern recognition receptors and pathogen effectors will be an important goal of future research.

Moreover, our study showed that plant biotrophs carry a lower number of CAZyme-encoding genes associated with plant cell wall degradation and apparently undergo a convergent loss of secondary metabolic gene clusters to trade off their sustainable parasitism. Spanu, et al. [41] discovered powdery mildew fungi that adapt to their specific biotrophy by increasing retrotransposons and reducing the number of enzymes involved in primary and secondary metabolism, carbohydrate-active enzymes, and transporters. Similarly, in comparison with hemibiotrophic *Phytophthora* species, Baxter, et al. [42] explored the oomycete *Hyaloperonospora arabidopsidis* (Hpa), an obligate biotroph, and reduced some genes encoding host-targeted, degradative enzymes and genes required for the synthesis of arachidonic acid and polyamine oxidases. Profiles derived from whole-genomic analysis also revealed that necrotrophic pathogens harbored an extensive set of CAZymes and secondary metabolic enzymes compared to biotrophic pathogens [35, 43]. All of these results demonstrated that biotrophs have evolved specific genetic features to accommodate their infection and survival strategy.

To address how variations in effectors have an impact on the interaction between hosts and pathogens, we investigated the genetic diversity of effector repertoires from different lifestyles. The diversity distribution analysis of effectors indicated clear separation of biotrophs from the other lifestyle groups (Fig. 3), consistent with biotrophs preferentially harboring distinct suites of effectors that have specific functions in biotrophic pathogenesis [41]. Moreover, a set of core effector families from pathogens with different lifestyles was observed in our study, which suggests that most pathogens defeat plant immunity with core conserved effectors. Even genome database-integrated sequences and annotations for dozens of fungi species have been established [44], most of them neglected effector candidate repertoire for plant pathogenic fungi. The genome database we established is complementary for the most devastating fungal plant pathogens, it allows researchers to compare the functional repertoire in the species used in this study and uncover novel effectors and their pathogenic mechanism.

## Conclusions

In this study, we presented a comparative genomic analysis of ascomycete plant pathogens and revealed genome variations among pathogenic fungi with different lifestyles. Plant ascomycete biotrophs appear to have very similar genomic characteristics, including much lower content in expanded or contracted gene families, genes coding for CAZymes, and enzymes participating in secondary metabolite synthesis. Their effector repertoire diversity shows remarkable differences relative to necrotrophs, hemibiotrophs and human pathogens. Although expanding the population genomics of plant pathogens offers a powerful approach to analyze whole-genome features and predict extensive effector gene repertoires, experimental verification of gene function in biological processes and effectors in virulence is still needed. Moreover, the variations of pathogen virulence in space and time are driven mainly by plant-pathogen co-evolutionary dynamics. Further research is in progress to try to understand more about virulence effectors evolution in plant immune system network.

## Methods

### Genome data collection

The fungi selected are among the most economically important groups of pathogens of native and cultivated plants. The members include the barley pathogens *B. graminis f.* sp. hordei and *Pyrenophora teres* f. teres, the wheat pathogens *F. graminearum*, *Gaeumannomyces tritici*, *Parastagonospora nodorum*, *Zymoseptoria tritici* and *Puccinia graminis* f. sp. tritici, the hemibiotrophic rice and wheat pathogen *M. oryzae*, the biotrophic maize pathogen *Ustilago maydis*, generalized economic crops and grass pathogens, and two human pathogens, *Aspergillus fumigatus* and *Histoplasma capsulatum* (Table S2). Genomes for all fungi were collected from the NCBI Assembly database (for assembly accessions, see Table S1) [45]. Three species of *B. graminis*, *Colletotrichum gloeosporioides* and *P. teres* f. teres lack annotation information, and their genome assemblies were each annotated using the MAKER Annotation Pipeline (http://gmod.org/wiki/MAKER_Tutorial) [46]. We detected and masked the repeats and transposable elements based on the algorithms GeneMark-ES [47] and SNAP [48] and obtained conceptually translated protein. Protein information of the closest homology for each sequence was retrieved from UniProt based on the best BLAST hit (maximum e-value of 1e^−5^). HMMER was performed to identify Pfam domains against the Pfam protein database [49].

### Phylogenomic analyses

These twenty-two genomes were subjected to phylogenomic analyses. The gene families with only one gene per species were identified by OrthoFinder [50]. A total of 531 single-copy gene families were produced (https://github.com/Wangys-prog/Fungal_effector_proteins/tree/main/Single_Copy_Orthologue_Sequences). For these families, we performed multiple sequence alignment and maximum likelihood (ML) tree estimation. Sequence alignment was performed in MAFFT [51] and HAlign [52, 53] with default settings. TrimAl was used to trim the alignment to eliminate poorly aligned regions [54]. The species tree was estimated in RAxML using the standard algorithm [55]. Visualization of the tree was performed with iTOL [56].

### Divergence date estimation

For divergence time evaluation for the genome-based tree, phylogeny was calibrated in r8s with the penalized-likelihood algorithm, and the smoothing parameter value was set to 1 through cross-validation [57]. Three calibration points were fixed in the molecular clock analysis. Node 1 was fixed to 635 Mya for the age estimates of Dikarya [16]. Node 2 was constrained to 500 - 582 Mya for the divergence of Basidiomycota [16, 58]. Node 3 was constrained to 207-339 Mya [59].

### Evolution of gene family sizes

The computational tool CAFE was used to analyze the gene families that have experienced expansion or contraction [60]. The program can estimate the evolution of gene family sizes over a phylogeny based on the stochastic birth and death model assuming that gene gain and loss are equally probable. The expansion or contraction events of gene families were estimated by the comparisons of family size differences between the most recent common ancestor (MRCA) and each of the current species with a significant *P*-value of 0.01. If the significance of gene family expansion in each branch was also less than 0.01 (*P*-value), these families were regarded as rapidly evolving families. The sequence count table of protein families from OrthoFinder and the ultrametric tree from r8s were used in the analyses.

### CAZymes annotation

The hmmscan program from the HMMER software package [61] was used to annotate the CAZyme domain by comparing query protein sequences to the dbCAN CAZyme domain HMM database (http://bcb.unl.edu/dbCAN2/download/Databases/) [62]. The dbCAN2 meta server grouped CAZyme functional modules into six classes: glycosyl transferases (GTs), glycoside hydrolases (GHs), polysaccharide lyases (PLs), carbohydrate esterases (CEs), enzymes for auxiliary activities (AAs) and carbohydrate-binding modules (CBMs) [63].

### Prediction of gene clusters for biosynthesis of specialized metabolites

Biosynthetic gene clusters (BGCs) for secondary metabolites were analyzed using antiSMASH, which applies a rule-based cluster detection approach to identify 45 different types of secondary metabolite biosynthetic pathways via their core biosynthetic enzymes. The 45 BGC classes were condensed into five major groups: nonribosomal peptide synthetase (NRPs), polyketide, ribosomally synthesized and posttranslationally modified peptides (RiPP), and terpenes [64].

### Prediction of the secretome and effectome

The SignalP 4.0 server was used initially to predict proteins with a signal peptide [65]. The TMHMM server was used to screen for predicted proteins without a predicted transmembrane domain (TMHMM server version 2.0; http://www.cbs.dtu.dk/services/TMHMM/). The putative effector proteins were predicted by EffectorP 2.0. The class probability threshold for the EffectorP classifier is set at 0.55 [66]. Secreted and effector protein sequences were clustered into orthologous families using MCL. Representative protein sequences were randomly selected from each family and used to calculate phylogenetic distance. The distance was visualized by principal coordinate plots using the “pcoa” function of R. ANOSIM analysis was used to statistically test whether there was a significant difference among groups [67]. The core and specific families were illustrated by Venn diagram and flower plot using VennDiagram and the plotrix package of R [68, 69].

### Genome database establishment

The web server was built using Drupal (http://www.drupal.org/), which is an open source platform that allows users to design robust and flexible websites. The framework and basic functionality of Drupal is written by PHP, and it supports many extensions, such as PHP, MySQL. Tripal provides other functions to extend the content of Drupal, such as genomic data loaders and data storage. Furthermore, Tripal also offers some extensions that support loading and visualization of BLAST and popular genome browsing tools, such as JBrowse [70].

## Supplementary Information

**Additional file 1.**

## Data Availability

All raw data is archived under NCBI BioProject and their accession numbers are available in Supplementary Table S1. The web-based genome browser of plant fungal pathogens is accessible at the http://lab.malab.cn/soft/PFPG. All the sequence data used can be downloaded from links on the home page.
