# Supplementary material for: Comparative genome analysis of plant ascomycete fungal pathogens with different lifestyles reveals distinctive virulence strategies
Source: BMC Genomics. 2022 Jan 7;23:34. doi: 10.1186/s12864-021-08165-1 (PMC8740420; doi:10.1186/s12864-021-08165-1)
Supplement: Supplementary file 1 — Additional file 1. [file 12864_2021_8165_MOESM1_ESM.docx]

**Supplementary Information**

1. **Supplementary Tables:**

**Table S1 Genomes included in this study**

**Table S2 Pathogen-host infection biology**

| Species | Strain | Host | Disease | References |
| --- | --- | --- | --- | --- |
| *Aspergillus fumigatus* | Af293 | Human | Human lung infection | Nierman, et al. [1] |
| *Botrytis cinerea* | B05.10 | Nonspecific | Gray mold rot | Van Kan, et al. [2] |
| *Blumeria graminis f.* sp. hordei | DH14 | Barley | Powdery mildew | Wu, et al. [3] |
| *Ceratocystis fimbriata* | CBS 114723 | Sweet potato | Sweet potato black rot | Simpson, et al. [4] |
| *Colletotrichum gloeosporioides* | SMCG1#C | Nonspecific | Anthracnose disease | Huang, et al. [5] |
| *Fusarium graminearum* | PH-1 | Wheat and barley | Fusarium head blight (FHB) | Cuomo, et al. [6] |
| *Fusarium oxysporum f.* sp. Lycopersici | 4287 | Tomato | Fusarium wilt of Tomato | Ma, et al. [7] |
| *Golovinomyces cichoracearum* | UMSG1 | Sow thistle | Powdery mildew | Wu, et al. [3] |
| *Gaeumannomyces tritici* | R3-111a-1 | Wheat | Take-all disease | Okagaki, et al. [8] |
| *Histoplasma capsulatum* | NAm1 | Mamalian | Mamalian infection | Kasuga, et al. [9] |
| *Leptosphaeria maculans* | JN3 | Oilseed rape | Blackleg of oilseed rape | Rouxel, et al. [10] |
| *Magnaporthe oryzae* | 70-15 | Rice | Rice blast | Dean, et al. [11] |
| *Parastagonospora nodorum* | SN15 | Wheat | Stagonospora nodorum blotch (SNB) | Hane, et al. [12] |
| *Pyrenophora seminiperda* | CCB06 | Grass | Seed pathogen | Soliai, et al. [13] |
| *Pyrenophora teres f. teres* | W1-1 | Barley | Barley net blotch | Syme, et al. [14] |
| *Sclerotinia sclerotiorum* | 1980 | Nonspecific | Sclerotinia rot | Amselem, et al. [15] |
| *Verticillium dahliae* | VdLs.17 | Nonspecific | Verticillium wilt | Klosterman, et al. [16] |
| *Venturia inaequalis* | EU-B04 | Apple | Apple scab | Le Cam, et al. [17] |
| *Zymoseptoria tritici* | IPO323 | Wheat | Septoria tritici blotch (STB) | Goodwin, et al. [18] |
| *Puccinia graminis f.* sp. tritici | CRL 75-36-700-3 | Wheat | Wheat stem rust | Duplessis, et al. [19] |
| *Melampsora larici-populina* | 98AG31 | Poplar | Poplar leaf rust | Duplessis, et al. [19] |
| *Ustilago maydis* | 521 | Maize | Maize Smut | Kämper, et al. [20] |

**Table S3 Gene family size changes of species**

| Species | Abbreviation | Expanded families | Rapidly expanded families | Genes gain | Contracted families | Rapidly contracted families | Genes loss |
| --- | --- | --- | --- | --- | --- | --- | --- |
| *Ustilago maydis* | Um | 188 | 10 | 289 | 1781 | 8 | 1866 |
| *Puccinia graminis f.* sp. tritici | Pg | 995 | 88 | 2533 | 609 | 56 | 703 |
| *Melampsora larici-populina* | Mlp | 737 | 83 | 2176 | 426 | 45 | 511 |
| *Histoplasma capsulatum* | Hc | 305 | 64 | 1201 | 1765 | 79 | 1918 |
| *Aspergillus fumigatus* | Af | 694 | 137 | 1081 | 515 | 22 | 562 |
| *Zymoseptoria tritici* | Zt | 641 | 84 | 1025 | 3640 | 5 | 3676 |
| *Venturia inaequalis* | Vi | 707 | 129 | 1337 | 2765 | 18 | 2805 |
| *Parastagonospora nodorum* | Pn | 858 | 224 | 1556 | 980 | 4 | 987 |
| *Leptosphaeria maculans* | Lm | 308 | 21 | 346 | 1649 | 63 | 1745 |
| *Pyrenophora seminiperda* | Ps | 206 | 100 | 247 | 2259 | 84 | 2387 |
| *Pyrenophora teres f. teres* | Pt | 444 | 276 | 1499 | 404 | 10 | 418 |
| *Blumeria graminis f.* sp. hordei | Bg | 200 | 36 | 690 | 515 | 6 | 522 |
| *Golovinomyces cichoracearum* | Gc | 119 | 27 | 361 | 406 | 10 | 429 |
| *Sclerotinia sclerotiorum* | Ss | 249 | 97 | 655 | 680 | 8 | 692 |
| *Botrytis cinerea* | Bc | 1522 | 513 | 2449 | 240 | 10 | 270 |
| *Gaeumannomyces tritici* | Gt | 729 | 156 | 1282 | 568 | 11 | 585 |
| *Magnaporthe oryzae* | Mo | 500 | 86 | 812 | 708 | 12 | 738 |
| *Ceratocystis fimbriata* | Cf | 97 | 21 | 328 | 5072 | 243 | 5632 |
| *Colletotrichum gloeosporioides* | Cg | 1289 | 473 | 3010 | 627 | 10 | 641 |
| *Verticillium dahliae* | Vd | 379 | 40 | 484 | 1740 | 67 | 1896 |
| *Fusarium oxysporum f.* sp. Lycopersici | Fo | 3485 | 1222 | 9699 | 286 | 1 | 287 |
| *Fusarium graminearum* | Fg | 237 | 67 | 303 | 1279 | 60 | 1380 |

**Table S4 Distribution of CAZyme-coding gene homologs in selected pathogenic fungi**

| Species | Abbreviation | GH | GT | PL | CE | AA | CBM |
| --- | --- | --- | --- | --- | --- | --- | --- |
| *Ustilago maydis* | Um | 117 | 96 | 4 | 67 | 46 | 4 |
| *Puccinia graminis f.* sp. tritici | Pg | 185 | 128 | 7 | 70 | 52 | 9 |
| *Melampsora larici-populina* | Mlp | 200 | 126 | 7 | 111 | 69 | 13 |
| *Histoplasma capsulatum* | Hc | 87 | 91 | 2 | 41 | 45 | 3 |
| *Aspergillus fumigatus* | Af | 282 | 138 | 21 | 107 | 80 | 13 |
| *Zymoseptoria tritici* | Zt | 206 | 135 | 4 | 98 | 82 | 12 |
| *Venturia inaequalis* | Vi | 239 | 123 | 17 | 151 | 124 | 12 |
| *Parastagonospora nodorum* | Pn | 291 | 135 | 13 | 167 | 191 | 26 |
| *Leptosphaeria maculans* | Lm | 231 | 126 | 20 | 119 | 113 | 10 |
| *Pyrenophora seminiperda* | Ps | 221 | 95 | 10 | 101 | 109 | 15 |
| *Pyrenophora teres f. teres* | Pt | 251 | 133 | 11 | 129 | 139 | 15 |
| *Blumeria graminis f.* sp. hordei | Bg | 75 | 85 | 1 | 35 | 20 | 1 |
| *Golovinomyces cichoracearum* | Gc | 65 | 85 | 2 | 30 | 20 | 1 |
| *Sclerotinia sclerotiorum* | Ss | 238 | 125 | 10 | 101 | 114 | 17 |
| *Botrytis cinerea* | Bc | 298 | 164 | 13 | 156 | 150 | 15 |
| *Gaeumannomyces tritici* | Gt | 276 | 135 | 9 | 121 | 162 | 28 |
| *Magnaporthe oryzae* | Mo | 277 | 137 | 8 | 139 | 171 | 31 |
| *Ceratocystis fimbriata* | Cf | 104 | 94 | 6 | 39 | 43 | 4 |
| *Colletotrichum gloeosporioides* | Cg | 457 | 160 | 47 | 251 | 274 | 32 |
| *Verticillium dahliae* | Vd | 290 | 123 | 39 | 125 | 138 | 12 |
| *Fusarium oxysporum f.* sp. Lycopersici | Fo | 484 | 275 | 29 | 271 | 260 | 82 |
| *Fusarium graminearum* | Fg | 269 | 148 | 24 | 152 | 146 | 25 |

**Table S5 Distribution of biosynthetic gene cluster in pathogenic fungi**

| Species | Abbreviation | NRPs | Polyketides | Terpenes |
| --- | --- | --- | --- | --- |
| *Ustilago maydis* | Um | 3 | 1 | 2 |
| *Puccinia graminis f.* sp. tritici | Pg | 1 | 0 | 4 |
| *Melampsora larici-populina* | Mlp | 1 | 0 | 3 |
| *Histoplasma capsulatum* | Hc | 4 | 1 | 4 |
| *Aspergillus fumigatus* | Af | 8 | 10 | 6 |
| *Zymoseptoria tritici* | Zt | 10 | 6 | 4 |
| *Venturia inaequalis* | Vi | 4 | 2 | 3 |
| *Parastagonospora nodorum* | Pn | 5 | 15 | 5 |
| *Leptosphaeria maculans* | Lm | 4 | 11 | 5 |
| *Pyrenophora seminiperda* | Ps | 7 | 8 | 5 |
| *Pyrenophora teres f. teres* | Pt | 72 | 11 | 4 |
| *Blumeria graminis f.* sp. hordei | Bg | 1 | 1 | 1 |
| *Golovinomyces cichoracearum* | Gc | 1 | 3 | 3 |
| *Sclerotinia sclerotiorum* | Ss | 6 | 15 | 3 |
| *Botrytis cinerea* | Bc | 6 | 15 | 9 |
| *Gaeumannomyces tritici* | Gt | 5 | 14 | 5 |
| *Magnaporthe oryzae* | Mo | 9 | 19 | 11 |
| *Ceratocystis fimbriata* | Cf | 2 | 3 | 2 |
| *Colletotrichum gloeosporioides* | Cg | 9 | 20 | 8 |
| *Verticillium dahliae* | Vd | 3 | 9 | 5 |
| *Fusarium oxysporum f.* sp. Lycopersici | Fo | 10 | 11 | 11 |
| *Fusarium graminearum* | Fg | 7 | 8 | 11 |

**Table S6 The biotrophs-specific effector families**

**Table S7 Pfam domains annotations of** **biotrophs-specific effector families**

| Target name | Sequence number | Domain accession | Descriptions |
| --- | --- | --- | --- |
| Hce2 | 5 | PF14856.6 | Pathogen effector; putative necrosis-inducing factor |
| Egh16-like | 3 | PF11327.8 | Egh16-like virulence factor |
| CFEM | 2 | PF05730.11 | CFEM domain |
| CM_2 | 2 | PF01817.21 | Chorismate mutase type |
| GILT | 2 | PF03227.16 | Gamma interferon inducible lysosomal thiol reductase |
| LOR | 2 | PF04525.12 | Family of unknow function (DUF5333) |
| SRP19 | 2 | PF01922.17 | SRP19 protein |
| Collagen_bind_2 | 1 | PF12904.7 | Putative collagen-binding domain of a collagenase |
| DUF1431 | 1 | PF07248.12 | Protein of unknown function(DUF1431) |
| DUF1856 | 1 | PF08983.10 | Domain of unknown function (DUF1856) |
| DUF333 | 1 | PF03891.15 | Domain of unknown function (DUF333) |
| DUF3724 | 1 | PF12521.8 | Protein of unknown function (DUF3724) |
| DUF4232 | 1 | PF14016.6 | Protein of unknown function (DUF4232) |
| DUF5333 | 1 | PF17267.2 | Family of unknow function (DUF5333) |
| GPI-anchored | 1 | PF10342.9 | Ser-Thr-rich glycosyl-phosphatidyl-inositol-anchored membrane family |
| Muted | 1 | PF14942.6 | Organelle biogenesis, Muted-like protein |
| PADR1 | 1 | PF08063.12 | PADR1 (NUC008) domain |
| Stig1 | 1 | PF04885.13 | Stigma-specific protein, Stig1 |
| Sugarporin_N | 1 | PF11471.8 | Maltoporin periplasmic N-terminal extension |
| TarH | 1 | PF02203.15 | Tar ligand binding domain homologue |
| YlbE | 1 | PF14003.6 | YlbE-like protein |
| Others | 62 |  | No annotation information |

**Table S8 The core effector families of** **phytopathogens with distinct lifestyles**

1. **Supplementary Figures :**


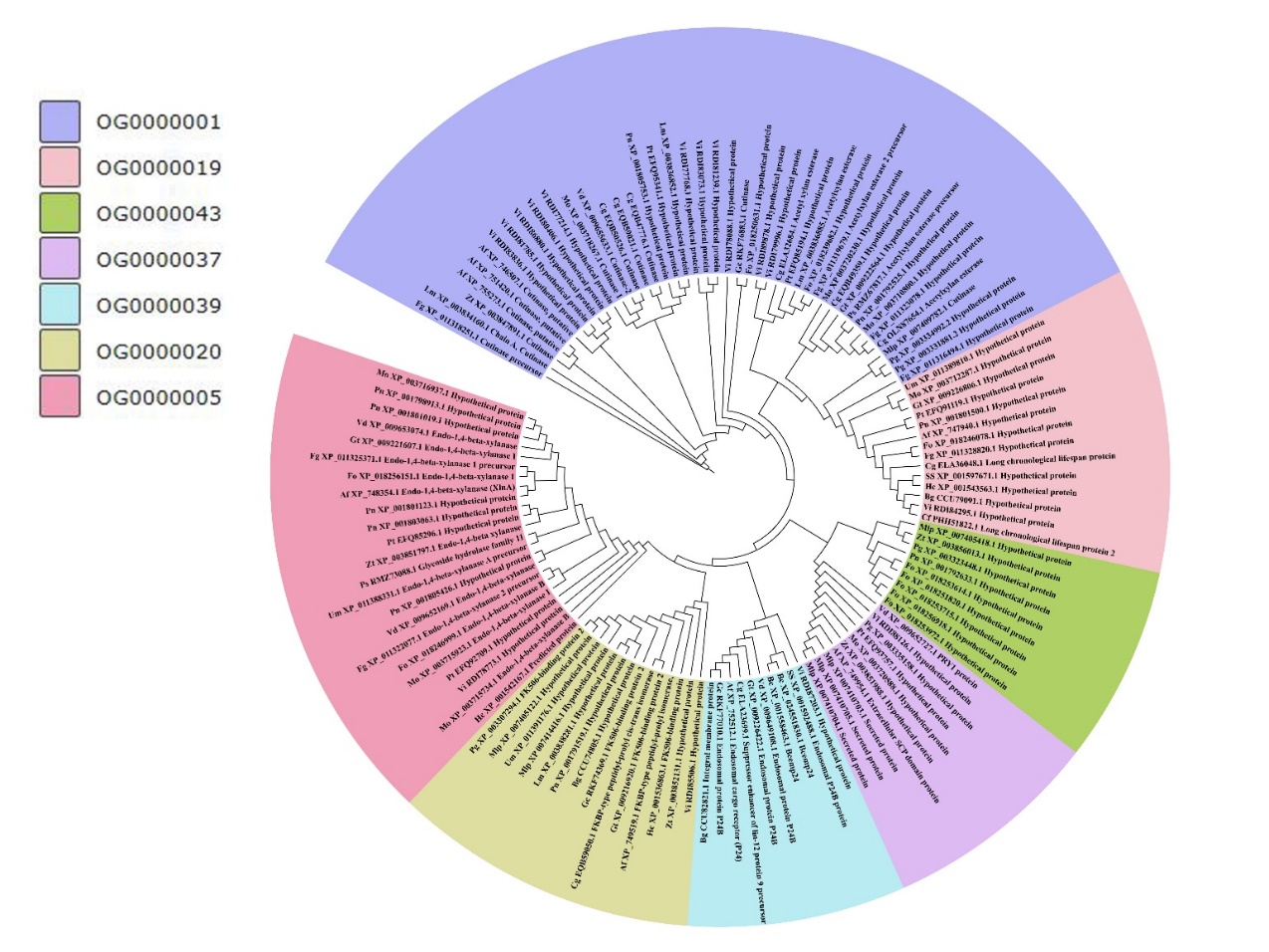


**Fig.S1 Phylogenetic analysis of core effector gene families**


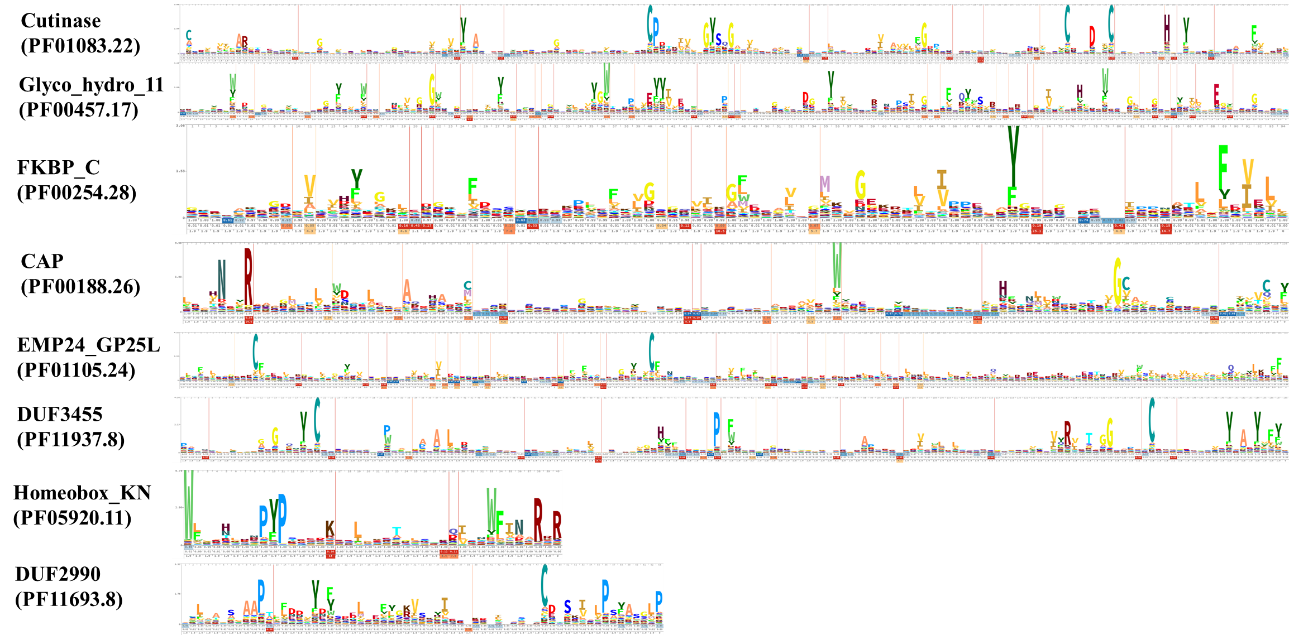


**Fig.S2 Domain visualization of** **core effector gene families**

**Reference**

1. Nierman WC, Pain A, Anderson MJ, Wortman JR, Kim HS, Arroyo J et al: **Genomic sequence of the pathogenic and allergenic filamentous fungus *Aspergillus fumigatus***. *Nature* 2005, **438**(7071):1151.

2. Van Kan JA, Stassen JH, Mosbach A, Van Der Lee TA, Faino L, Farmer AD et al: **A gapless genome sequence of the fungus *Botrytis cinerea***. *Molecular plant pathology* 2017, **18**(1):75-89.

3. Wu Y, Ma X, Pan Z, Kale SD, Song Y, King H et al: **Comparative genome analyses reveal sequence features reflecting distinct modes of host-adaptation between dicot and monocot powdery mildew**. *BMC Genomics* 2018, **19**(1):705.

4. Simpson MC, Wilken PM, Coetzee MP, Wingfield MJ, Wingfield BD: **Analysis of microsatellite markers in the genome of the plant pathogen *Ceratocystis fimbriata***. *Fungal biology* 2013, **117**(7-8):545-555.

5. Huang L, Kim K-T, Yang J-Y, Song H, Choi G, Jeon J et al: **A high-quality draft genome sequence of *Colletotrichum gloeosporioides* sensu stricto SMCG1# C, a causal agent of anthracnose on *Cunninghamia lanceolata* in China**. *Molecular plant-microbe interactions* 2019, **32**(2):139-141.

6. Cuomo CA, Güldener U, Xu J-R, Trail F, Turgeon BG, Di Pietro A et al: **The *Fusarium graminearum* genome reveals a link between localized polymorphism and pathogen specialization**. *Science* 2007, **317**(5843):1400-1402.

7. Ma L-J, Van Der Does HC, Borkovich KA, Coleman JJ, Daboussi M-J, Di Pietro A et al: **Comparative genomics reveals mobile pathogenicity chromosomes in *Fusarium***. *Nature* 2010, **464**(7287):367-373.

8. Okagaki LH, Nunes CC, Sailsbery J, Clay B, Brown D, John T et al: **Genome sequences of three phytopathogenic species of the Magnaporthaceae family of fungi**. *G3: Genes, Genomes, Genetics* 2015, **5**(12):2539-2545.

9. Kasuga T, White TJ, Koenig G, Mcewen J, Restrepo A, Castañeda E et al: **Phylogeography of the fungal pathogen *Histoplasma capsulatum***. *Molecular ecology* 2003, **12**(12):3383-3401.

10. Rouxel T, Grandaubert J, Hane JK, Hoede C, Van de Wouw AP, Couloux A et al: **Effector diversification within compartments of the *Leptosphaeria maculans* genome affected by repeat-induced point mutations**. *Nature communications* 2011, **2**(1):1-10.

11. Dean RA, Talbot NJ, Ebbole DJ, Farman ML, Mitchell TK, Orbach MJ et al: **The genome sequence of the rice blast fungus *Magnaporthe grisea***. *Nature* 2005, **434**(7036):980-986.

12. Hane JK, Lowe RG, Solomon PS, Tan K-C, Schoch CL, Spatafora JW et al: **Dothideomycete–plant interactions illuminated by genome sequencing and EST analysis of the wheat pathogen *Stagonospora nodorum***. *Plant Cell* 2007, **19**(11):3347-3368.

13. Soliai MM, Meyer SE, Udall JA, Elzinga DE, Hermansen RA, Bodily PM, Hart AA, Coleman CE: **De novo genome assembly of the fungal plant pathogen *Pyrenophora semeniperda***. *PLoS One* 2014, **9**(1).

14. Syme RA, Martin A, Wyatt NA, Lawrence JA, Muria-Gonzalez MJ, Friesen TL, Ellwood SR: **Transposable element genomic fissuring in *Pyrenophora teres* is associated with genome expansion and dynamics of host–pathogen genetic interactions**. *Frontiers in Genetics* 2018, **9**:130.

15. Amselem J, Cuomo CA, van Kan JA, Viaud M, Benito EP, Couloux A et al: **Genomic analysis of the necrotrophic fungal pathogens *Sclerotinia sclerotiorum* and *Botrytis cinerea***. *PLoS genetics* 2011, **7**(8):e1002230.

16. Klosterman SJ, Subbarao KV, Kang S, Veronese P, Gold SE, Thomma BP et al: **Comparative genomics yields insights into niche adaptation of plant vascular wilt pathogens**. *PLoS pathogens* 2011, **7**(7).

17. Le Cam B, Sargent D, Gouzy J, Amselem J, Bellanger M-N, Bouchez O et al: **Population genome sequencing of the scab fungal species *Venturia inaequalis*, *Venturia pirina*, *Venturia aucupariae* and *Venturia asperata***. *G3: Genes, Genomes, Genetics* 2019, **9**(8):2405-2414.

18. Goodwin SB, M'Barek SB, Dhillon B, Wittenberg AH, Crane CF, Hane JK et al: **Finished genome of the fungal wheat pathogen *Mycosphaerella graminicola* reveals dispensome structure, chromosome plasticity, and stealth pathogenesis**. *PLoS genetics* 2011, **7**(6).

19. Duplessis S, Cuomo CA, Lin Y-C, Aerts A, Tisserant E, Veneault-Fourrey C et al: **Obligate biotrophy features unraveled by the genomic analysis of rust fungi**. *Proceedings of the National Academy of Sciences* 2011, **108**(22):9166-9171.

20. Kämper J, Kahmann R, Bölker M, Ma L-J, Brefort T, Saville BJ et al: **Insights from the genome of the biotrophic fungal plant pathogen *Ustilago maydis***. *Nature* 2006, **444**(7115):97-101.
